# Job Flows Into and Out of Health Care Before and After the COVID-19 Pandemic

**DOI:** 10.1001/jamahealthforum.2023.4964

**Published:** 2024-01-26

**Authors:** Karen Shen, Julia C.P. Eddelbuettel, Matthew D. Eisenberg

**Affiliations:** 1 Department of Health Policy and Management, Johns Hopkins Bloomberg School of Public Health, Baltimore, Maryland; 2 PhD Program in Health Policy, Harvard University, Boston, Massachusetts

## Abstract

**Importance:**

Anecdotal evidence suggests that health care employers have faced increased difficulty recruiting and retaining staff in the wake of the COVID-19 pandemic. Empirical research is needed to understand the magnitude and persistence of these changes, and whether they have disproportionate implications for certain types of workers or regions of the country.

**Objective:**

To quantify the number of workers exiting from and entering into the health care workforce before and after the pandemic and to examine variations over time and across states and worker demographics.

**Design, Setting, and Participants:**

This cohort study used US Census Bureau state unemployment insurance data on job-to-job flows in the continental US to construct state-level quarterly exit and entry rates for the health care industry from January 2018 through December 2021 (Arkansas, Mississippi, and Tennessee were omitted due to missing data). An event study design was used to compute quarterly mean adjusted rates of job exit from and entry into the health care sector as defined by the North American Industry Classification System. Data were examined from January to June 2023.

**Exposure:**

The COVID-19 pandemic.

**Main Outcomes and Measures:**

The main outcomes were the mean adjusted health care worker exit and entry rates in each quarter by state and by worker demographics (age, gender, race and ethnicity, and education level).

**Results:**

In quarter 1 of 2020, there were approximately 18.8 million people (14.6 million females [77.6%]) working in the health care sector in our sample. The exit rate for health care workers increased at the onset of the pandemic, from a baseline quarterly mean of 5.9 percentage points in 2018 to 8.0 (95% CI, 7.7-8.3) percentage points in quarter 1 of 2020. Exit rates remained higher than baseline levels through quarter 4 of 2021, when the health care exit rate was 7.7 (95% CI, 7.4-7.9) percentage points higher than the 2018 baseline. In quarter 1 of 2020, the increase in health care worker exit rates was dominated by an increase in workers exiting to nonemployment (78% increase compared with baseline); in contrast, by quarter 4 of 2021, the exit rate was dominated by workers exiting to employment in non–health care sectors (38% increase compared with baseline). Entry rates into health care also increased in the postpandemic period, from 6.2 percentage points at baseline to 7.7 percentage points (95% CI, 7.4-7.9 percentage points) in the last quarter of 2021, suggesting increased turnover of health care staff. Compared with prepandemic job flows, the share of workers exiting health care after the pandemic who were female was disproportionately larger, and the shares of workers entering health care who were female or Black was disproportionately smaller.

**Conclusions and Relevance:**

Results of this cohort study suggest a substantial and persistent increase in health care workforce turnover after the pandemic, which may have long-lasting implications for workers’ willingness to remain in health care jobs. Policymakers and health care organizations may need to act to prevent further losses of experienced staff.

## Key Points

**Question:**

How did the COVID-19 pandemic affect entries into and exits out of the health care workforce?

**Findings:**

In this cohort study of approximately 18 million health care industry employees, the number of workers exiting from the industry peaked in the first quarter of 2020 but was elevated above 2018 baseline levels in all quarters from 2020 through 2021. In early 2020, exits were primarily from workers exiting to nonemployment, while in late 2021, exits were primarily from workers exiting to other sectors.

**Meaning:**

The findings of this study suggest a substantial and persistent increase in health care workforce turnover after the pandemic, which suggests the pandemic may have long-lasting implications for workers' willingness to remain in health care jobs.

## Teaser

This cohort study uses US Census data to examine changes in job flow rates into and out of the health care sector before and after the COVID-19 pandemic.

## Introduction

Employers in all economic sectors have reported that the COVID-19 pandemic created challenges to their ability to hire and retain workers.^1,2,3^ These challenges may have been exacerbated in the health care sector, where workers faced unique risks and responsibilities and have reported high rates of burnout.^4^ While there is concern that these conditions may have contributed to workers leaving the health care workforce in large numbers,^5^ evidence suggests that with the exception of a few settings (skilled nursing facilities and inpatient or residential behavioral health facilities), health care employment levels had largely returned to baseline values by the end of 2020.^6,7^ However, even in the absence of changes in employment levels, the pandemic may be associated with other health care workforce patterns that can be meaningful to patients and health care organizations. For example, there has been an increase in the use of staffing agencies to address staff shortages since the beginning of the pandemic, which may affect quality of care.^8,9,10,11^ Another important dimension is workforce turnover or churn. An increase in turnover may be associated with poorer patient outcomes^12,13^ by disrupting continuity of care and by diminishing the years of experience of the workforce. Also, turnover strains the firms employing the health care worker due to the need to devote more resources to recruiting and training workers.^14^ It is possible that health care worker turnover may have increased over the course of the pandemic even if employment levels remained relatively stable. Indeed, research using population survey data has found an increase in health care worker turnover after the pandemic, particularly among women with young children,^15^ but due to sample size constraints in that study, more precise estimates of the dynamics and geography of turnover are unknown. Understanding whether turnover disproportionately affects different areas of the country, different types of workers, or different types of firms could provide important evidence on the types of policies that are most needed to support the health care workforce.^16^

The present study seeks to fill this gap by examining the implications of the pandemic for turnover of the health care workforce using job flows data. While used more broadly in macroeconomic studies,^17,18^ the administrative job flows data that we used have been underused in health care workforce research. These data capture almost all job flows in the US on a quarterly basis, allowing us to examine the evolution of exit and entry rates of health care workers over the course of the pandemic. The data also contain additional information on the industries workers are exiting to or entering from, the states these workers live in, and the demographic makeup of the exiting and entering workers.

We used these data and an event study design to assess how turnover of the health care workforce changed after the COVID-19 pandemic, whether turnover was associated with job flows into nonemployment or into other workforce sectors, and whether specific states or demographic populations were disproportionately affected by these pandemic forces. These questions are important as policymakers consider what is most needed to support the health care workforce. If health care workforce turnover has indeed increased, policymakers should consider policies that are specifically targeted at reducing turnover, such as improving health care worker mental health, working conditions, wages, and benefits. Understanding whether health care workers are leaving for other employment sectors or for nonemployment can also inform whether the underlying factors associated with turnover are more characteristic of the changing competitiveness of the health care sector in the labor market or of increased barriers to labor force participation, such as a person’s caregiving obligations. Finally, understanding the demographics of workers who have been most affected is critical to ensure a diverse workforce that is equipped to meet the needs of diverse populations.^19,20^

## Methods

Per Johns Hopkins Institutional Review Board policy, this study was exempt from review because it was not human subjects research and the data used were deidentified and publicly available. We followed the Strengthening the Reporting of Observational Studies in Epidemiology (STROBE) reporting guideline for cross-sectional studies.

### Data Source

The primary data source was the Job-to-Job Flows (J2J) data,^21^ which are produced by the US Census Bureau from state administrative data, primarily state unemployment insurance data. These data include all workers who are covered by state unemployment insurance, totaling more than 95% of all US workers (major exclusions include federal government workers and independent contractors). The J2J data files use these unemployment insurance data to count job flow, defined as a change in main employer in a given quarter relative to the previous quarter, and area. While the underlying data are microdata, the publicly available data are available only in aggregated form. Specifically, the data are available for each origin-destination industry pair, where industries are defined at the 2-digit North American Industry Classification System (NAICS) level. These counts of industry-to-industry transitions are available at the metropolitan statistical area, state, and national levels and by worker demographics (age, sex [female or male], race [Black, White, or other (including Asian, Native Hawaiian or Other Pacific Islander, or multiracial), ethnicity [Hispanic or Latino or not Hispanic or Latino], educational level), and firm characteristics (size and age). We used state-level data from quarter 1 of 2018 through quarter 4 of 2021 for the continental US (ie, excluding Alaska and Hawaii because of missing data); data from Washington, DC, were included but 3 states in the continental US (Arkansas, Mississippi, and Tennessee) did not report data for the entirety of our sample period and were thus omitted from the analysis.

### Statistical Analysis

We leveraged these data to create industry-level exit and entry rates for each quarter. Our analysis focused on flows into and out of NAICS sector 62,^22^ which corresponds with health care and social assistance employers, and which we refer to as the health care sector. eTable 1 in Supplement 1 lists the distribution of employment across more granular industry categories within this sector. The primary variable of interest was the health care exit rate, which was defined as the share of all health care workers relative to all industry workers within a quarter who left the health care industry in that quarter. The numerator of this variable was defined as the sum of all workers who left a health care job for nonemployment (ie, people who had no end-of-quarter main job with any health care firm) and all workers who left a health care job for a job in a non–health care sector. We did not count workers who moved jobs within NAICS sector 62. We also considered entry rate, which was defined as the share of all health care workers relative to all industry workers within a quarter who started their job in that quarter. We separated exit and entry rates into workers who exited into (ie, entered from) nonemployment, and those who exited into (ie, entered from) a job in a different sector. Additional details on the construction of these measures are provided in the eAppendix in Supplement 1.

We used an event study design to compute adjusted mean exit and entry rates in each quarter. To do this, we used regression models at the state-quarter level that adjusted for state-level and seasonal fixed effects, and then used the coefficients from these regression models to compute the adjusted overall mean exit and entry rates for each quarter. The SEs were clustered at the state level, and the adjusted rates were computed using 2018 as the baseline year. The focus was on the postpandemic period between quarter 1 of 2020 and quarter 4 of 2021; we also computed adjusted rates for 2019 for comparison purposes. We described this methodology in detail in the eAppendix in Supplement 1. As we are interested in exit and entrance rates, we did not include additional covariates in these models.

We used these event studies to show the adjusted mean exit rates by quarter for the health care sector from January 2019 to December 2021 (2018 is omitted because it was used as the baseline year), reporting values as percentage point differences of the share of health care workers relative to all industry workers. We then showed health care exit rates decomposed by whether the worker exited into nonemployment or into a job in a non–health care sector. We then performed the same analysis for entry rates. Finally, we described whether the pandemic disproportionately affected certain states and certain types of workers. To assess geographic differences, we calculated the difference in the exit rate by state in each year of the pandemic (2020 and 2021) and prepandemic periods (2018-2019) and produced maps of these differences. To assess changes in the demographic compositions of exiters and entrants, we calculated the difference in the share of workers who exited or entered the health care sector and belonged to different demographic groups in the pandemic period relative to the prepandemic period. We also examined whether the patterns differed according to worker education level, employer firm size, and firm age to better understand the generalizability of our results. We performed the data analysis from January to June 2023 using Stata MP, version 17.0 (StataCorp, LLC).

## Results

**Figure 1.**
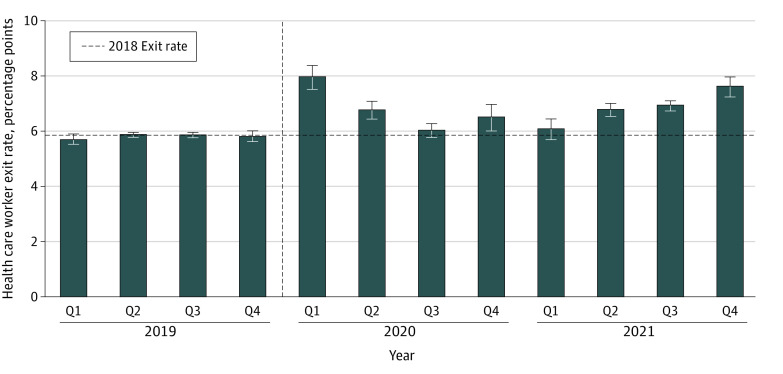
Adjusted Mean Quarterly Exit Rates From the Health Care Sector Over Time Estimated values from regression analyses of exit rates from the health care sector per quarter (Q), controlling for seasonal fixed effects and state fixed effects. Data from Q1 of 2018 through Q4 of 2021 for 45 states and Washington, DC, were used (Alaska, Arkansas, Hawaii, Mississippi, and Tennessee were omitted due to missing data). Estimated values represent the mean deviation per state from baseline (ie, the 2018 mean exit rate for health care workers) as a share of exits for workers in all industries. A health care worker exit in a quarter was defined as a person who started the quarter in a health care industry job and was not working in the health care industry by the end of the quarter. Error bars represent 95% CIs. The vertical line represents the onset of the COVID-19 pandemic.

In quarter 1 of 2020, there were approximately 18.8 million people (14.6 million females [77.6%] and 4.2 million males [22.4%]) working in the health care sector in our sample (eTable 2 in Supplement 1). Compared with the 2018 mean health care worker exit rate of 5.9 percentage points per quarter, we found that health care exit rates were stable in 2019 but then increased significantly in the first quarter of the pandemic (ie, quarter 1 of 2020) to 8.0 (95% CI, 7.7-8.3) percentage points (Figure 1). These exit rates remained higher than the 2018 baseline level through the end of the study period and were increasing at the end of 2021 to levels close to those observed in early 2020: 7.7 (95% CI, 7.4-7.9) percentage points in quarter 4 of 2021. eFigure 1 in Supplement 1 compares these patterns to the mean exit rate for non–health care sectors. We found a similar increase in industry exits in quarter 1 of 2020 for the non–health care sector, but in contrast to the health care sector, non–health care exit rates on average returned to near 2018 baseline levels for the remainder of the study period.

**Figure 2.**
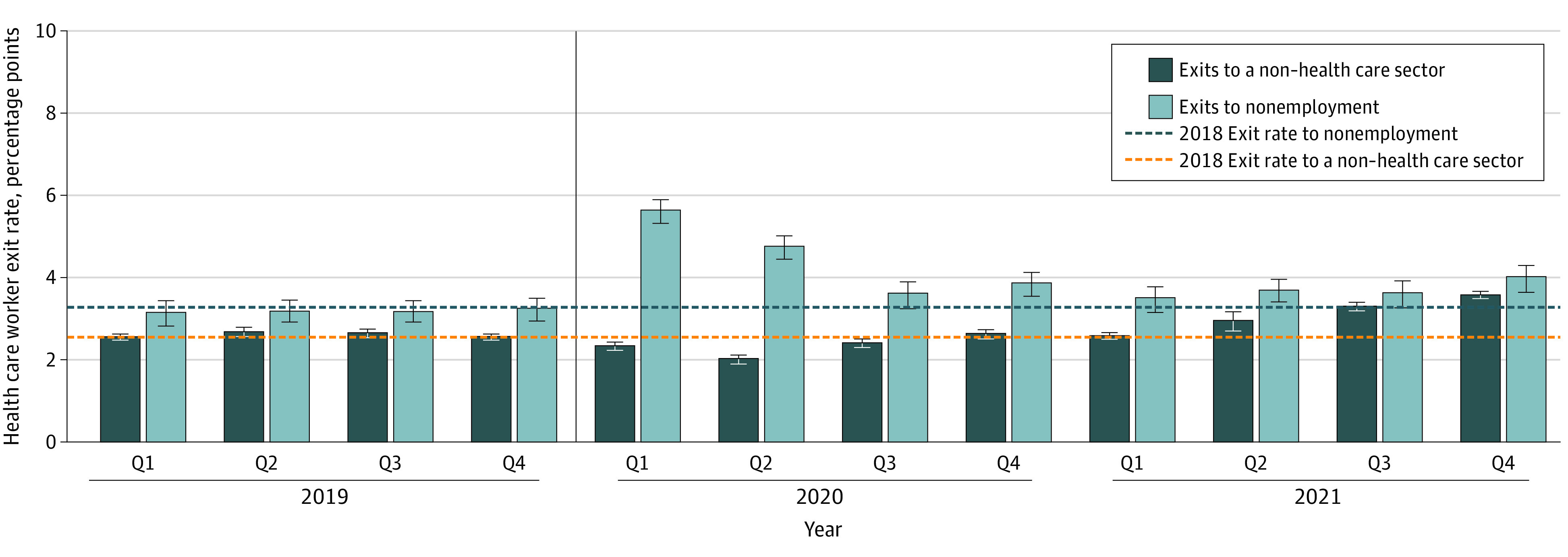
Adjusted Mean Exit Rates From the Health Care Sector Over Time by Exit Type Estimated values from regression analyses of exit rates from the health care sector per quarter (Q), controlling for seasonal fixed effects and state fixed effects. Data from Q1 of 2018 through Q4 of 2021 for 45 states and Washington, DC, were used (Alaska, Arkansas, Hawaii, Mississippi, and Tennessee were omitted due to missing data). Estimated values represent the mean deviation per state from baseline (ie, the 2018 mean exit rate for health care workers) as a share of exits for workers in all industries. A health care exit in a quarter is defined as someone who started the quarter in a health care industry job and was not working in the health care industry by the end of the quarter. A health care exit to nonemployment is defined as someone who did not work any job in the relevant quarter and the next quarter. A health care exit to a non–health care sector is an exiter who began a new job in a different sector in the relevant quarter or the next quarter. Error bars represent 95% CIs. The vertical line represents the onset of the COVID-19 pandemic.

Figure 2 shows the decomposition of the health care worker exit rate into people who left for nonemployment and people who left for jobs in other sectors. We found that the increase in health care worker exit rates in early 2020 was due to an increase in people exiting to nonemployment. Of the 8.0 (95% CI, 7.7-8.3) percentage point increase in people who exited the health care sector in quarter 1 of 2020, 5.7 percentage points were attributable to people exiting into nonemployment compared with a baseline mean of 3.2 percentage points per quarter in 2018, a 78% increase. On the other hand, a smaller share of the health care workforce exited into jobs in other sectors in quarter 1 of 2020 than in 2018. We estimated a 23% decline in the exit rate of the health care workforce into other sectors in quarter 1 of 2020, from a 2018 baseline mean of 2.6 percentage points to 2.0 (95% CI, 2.0-2.1) percentage points. These patterns were significantly different than those observed later in the pandemic: over time, the health care worker exit rate into other sectors increased and began to exceed baseline levels in quarter 2 of 2021. In quarter 4 of 2021, the last quarter of the sample, the exit rate of health care workers into nonemployment was 4.0 (95% CI, 3.8-4.3) percentage points, a 25% increase from baseline, and the exit rate into a non–health care sector was 3.6 (95% CI, 3.5-3.7) percentage points, a 38% increase from baseline.

**Figure 3.**
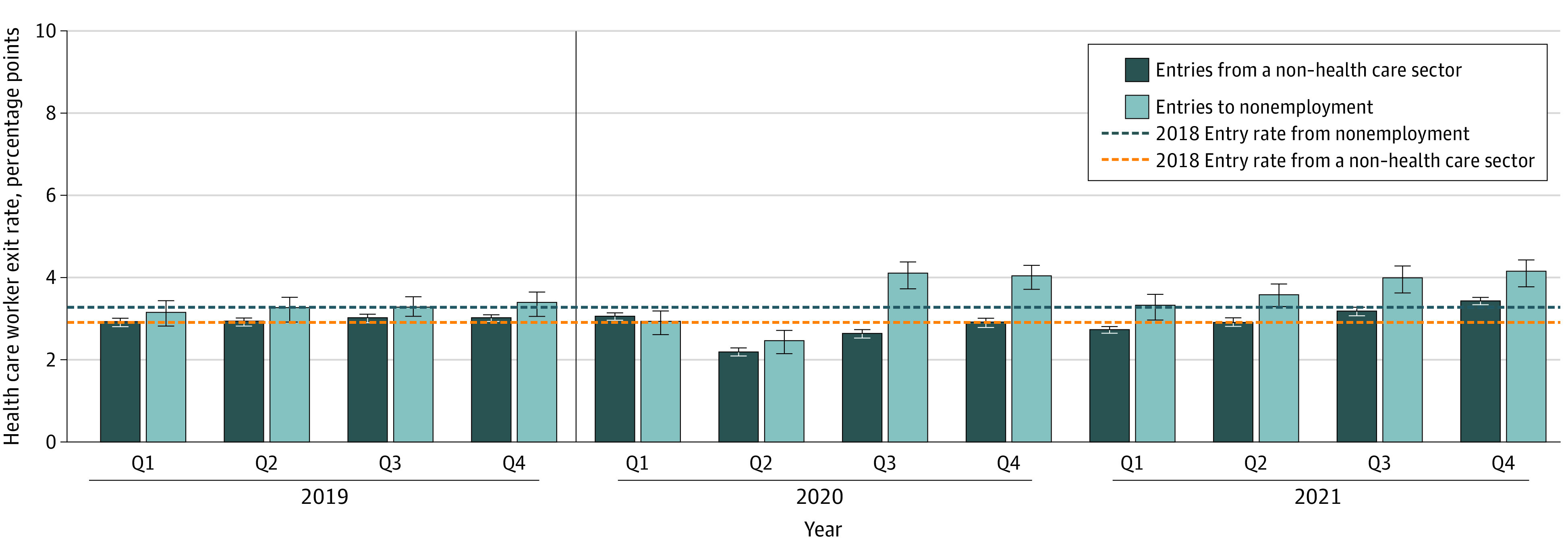
Adjusted Mean Entry Rates to the Health Care Sector Over Time by Entry Type Estimated values from regression analyses of entry rates into the health care sector per quarter (Q), controlling for seasonal fixed effects and state fixed effects. Data from Q1 of 2018 through Q4 of 2021 for 45 states and Washington, DC, were used (Alaska, Arkansas, Hawaii, Mississippi, and Tennessee were omitted due to missing data). Estimated values represent the mean deviation per state from baseline (ie, the 2018 mean entry rate) as a share of entries for workers in all industries. A health care entrant in a quarter is defined as someone who was not working in the health care industry at the beginning of the quarter, but was working in the health care industry at the end of the quarter. Health care entrants from nonemployment are people who had a full quarter of nonemployment prior to the reference quarter. Health care entrants from non–health care sectors were people who were working a job in a different sector in the previous quarter. Error bars represent 95% CIs. The vertical line represents the onset of the COVID-19 pandemic.

Figure 3 shows the ability of health care industries to address the increased exit rates with increased hiring by considering the evolution of entry rates into health care over the course of the pandemic. Entry rates into health care from both nonemployment and other sectors decreased slightly relative to baseline in quarters 1 and 2 of 2020, indicating that in these quarters, health care employers faced both higher exit rates and lower entry rates. However, since quarter 3 of 2020, entry rates into health care have exceeded the 2018 baseline and are of a similar magnitude to exit rates, suggesting that health care employers were able to make up some of the increase in exits with additional hires in late 2021. Compared with baseline exit and entry rates of 5.9 and 6.2 percentage points, at the end of 2021 the total exit and entry rates were 7.7 (95% CI, 7.4-7.9) percentage points and 7.6 (95% CI, 3.9-4.4) percentage points, respectively. While the increase in entrants means that total employment did not decrease by as much as the increase in exits alone would suggest, it implies that health care organizations after the pandemic are operating with more staff with less experience than in the prepandemic period. In particular, entry from nonemployment has been higher than baseline in most quarters beginning in quarter 3 of 2020. Compared with baseline levels, entry of new hires from nonemployment (3.3 percentage points vs 4.2 [95% CI, 3.9 to 4.4] percentage points) and from non–health care sectors (2.9 percentage points vs 3.4 [95% CI, 3.4-3.5] percentage points) was higher at the end of 2021. These rates correspond to a 27% increase in entries from nonemployment and a 17% increase in the entry rate from non–health care sectors.

**Figure 4.**
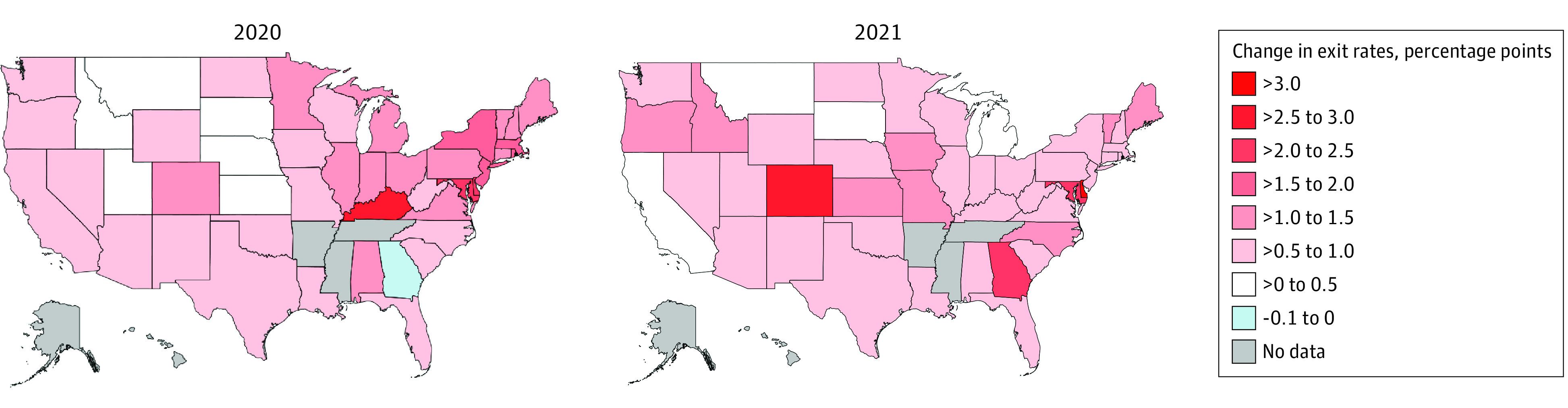
Change in Health Care Sector Exit Rates Between Prepandemic (2018-2019) and Postpandemic Periods (2020 and 2021) by State Data from quarter 1 of 2018 through quarter 4 of 2021 for 45 states and Washington, DC, were used (Alaska, Arkansas, Hawaii, Mississippi, and Tennessee were omitted due to missing data). A health care exit in a quarter is defined as someone who started the quarter in a health care industry job and was not working in the health care industry by the end of the quarter.

Figure 4 shows the geographic differences in health care worker exit rates between the prepandemic (2018-2019) and postpandemic periods (2020-2021). We calculated the differences separately for 2020 and 2021. Almost all states experienced an increase in turnover after the pandemic. In 2020, states in the Northeast region saw the greatest increases in health care worker exit rates vs their prepandemic mean values, comprising 8 of the top 10 states with the largest increases in health care worker exit rates. For example, in 2020, Delaware, Maryland, and New Jersey experienced elevated exit rate increases of 2.4, 2.3, and 2.0 percentage points, respectively, relative to prepandemic values. In 2021, the states with the greatest health care worker exit rates included more states in the South and West. For example, the 2021 health care worker exit rates in Colorado and Georgia were 2.7 and 2.5 percentage points higher than baseline, respectively. Health care worker exit rates for each state in each period are provided in eTable 3 in Supplement 1.

**Figure 5.**
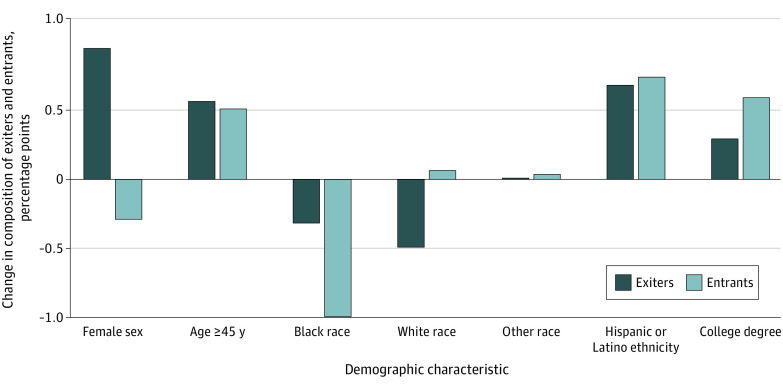
Change in Demographic Composition of Health Care Sector Exiters and Entrants Between Prepandemic (2018-2019) and Postpandemic Periods (2020-2021) Differences in the share of all health care sector exiters and entrants belonging to different demographic groups from quarter 1 of 2020 through quarter 4 of 2021, compared with the share of health care sector exiters and entrants belonging to those demographic groups from quarter 1 2018 through quarter 4 2019. Data for 45 states and Washington, DC, were used (Alaska, Arkansas, Hawaii, Mississippi, and Tennessee were omitted due to missing data). A health care exit in a quarter is defined as someone who started the quarter in a health care industry job and was not working in the health care industry by the end of the quarter. A health care entrant in a quarter is defined as someone who was not working in the health care industry at the beginning of the quarter, but was working in the health care industry at the end of the quarter. Other race includes Asian, Native Hawaiian or Other Pacific Islander, or multiracial individuals.

Figure 5 shows how the demographic profile of workers who exited and entered health care changed after the pandemic through the difference in demographic characteristics in the postpandemic period compared with the prepandemic period. The share of health care workers who exited the sector and were female increased by 0.95 percentage points during the pandemic, while the share of workers entering into the health care sector who were female followed an opposite pattern, decreasing by 0.30 percentage points. Together, these 2 results suggest a net loss of female health care workers after the pandemic. We also found evidence of a net loss in Black workers, with a 1.01–percentage point decline in the share of Black entrants (the share of Black exiters also declined in this period, but by a smaller amount, 0.32 percentage points). We also examined patterns by age, educational level, and race and ethnicity. We found that older workers were more likely to both exit and enter the health care workforce in the pandemic period, when the share of health care exiters and entrants who were aged 45 years or older increased by 0.56 and 0.51 percentage points, respectively. Similarly, we found that workers with a college education were disproportionately represented among both pandemic exiters and pandemic entrants, with the share of health care exiters and entrants with a college education increasing by 0.29 and 0.58 percentage points, respectively. These results suggest increasing churn among these workers after the pandemic, but not a statistically significant net change in the workforce composition of these workers.

To understand the generalizability of the findings, we also assessed whether the main patterns observed differ across worker educational level, firm size, and firm age (eFigures 2-4 in Supplement 1). We found similar patterns by worker educational level, suggesting that the observed patterns were somewhat common across occupations. We found a much larger increase in exiters in quarter 1 of 2020 for small firms, while large firms contributed to the late 2021 increase in health care exiters. Finally, we found that the measured health care exit rates were similar when we restricted analyses to firms that were 5 years old or older, while the patterns for younger firms were less clear.

## Discussion

This cohort study found a significant increase in health care worker turnover after the COVID-19 pandemic. In particular, we found that even though overall employment levels broadly stabilized in the health care sector by the end of 2020,^6^ health care worker exit rates remained elevated above 2018 baseline levels through the end of 2021. These increased exit rates were matched in part by an increase in hiring by health care firms during the pandemic, which helps explain why employment levels were not as affected as turnover rates. The increase in health care workforce turnover may pose substantial costs for both organizations and patients, as it implies potentially disrupted continuity of care and fewer staff with industry- and firm-specific experience. Increasing evidence has suggested that staff dissatisfaction and staff turnover in health care settings can have unfavorable implications for patient care even without staffing shortages.^12,23,24,25^

There has been concern that the implications of the pandemic for the health care workforce may be long-lasting, as workers who stayed employed through the stress of the pandemic leave for new opportunities now. In addition, the robust labor market may have intensified these exits. We found that the composition of these additional exits shifted over time from being primarily associated with additional health care workers exiting into nonemployment in early 2020 to more health care workers exiting to other, non–health care sectors in 2021. This increase in people exiting to other sectors may be a cause for concern for health care organizations and policymakers, as it may suggest a declining overall competitiveness of health care jobs in the broader labor market. To address this, policymakers and health care organizations may consider policies to improve working conditions through establishing staffing ratios, supporting career development, and offering support for dependent care.^5^

We found evidence of greater changes after the pandemic for female and Black health care workers. Specifically, results of the present study suggest that the population exiting health care in 2020 through 2021 included substantially more female workers than the population who exited health care in the prepandemic period, which is consistent with other reported evidence on how the pandemic affected female workers.^15^ Results of the present study also suggest that Black workers composed a lower fraction of entrants during the postpandemic period than during the prepandemic period, implying that health care employers’ ability to recruit Black workers after the pandemic may have been reduced. It is well known that female and Black health care workers are disproportionately concentrated in direct care occupations, which are paid the lowest wages and faced the greatest risks during the pandemic, and so it is unsurprising that these workers appear to be disproportionately less likely to be returning to or choosing to enter the health care workforce. At the same time, these patterns are likely to exacerbate preexisting diversity issues within the health care workforce. In particular, it is widely acknowledged that addressing the shortage of Black health care workers could have meaningful implications for the care of Black patients. As employers and policymakers plan strategies to help the health care industry recover from the pandemic, results of the present study suggest that efforts to specifically improve the recruitment of Black health care workers may be even more important. These efforts should work alongside policies that aim to broadly improve wages and working conditions for direct care workers and other occupations that disproportionately employ marginalized racial and ethnic groups.

We also found that the additional exits from the health care sector did not occur in all regions of the US equally over time. While states in the Northeast saw the greatest increases in health care worker exit rates in 2020, states in the South and West saw greater increases in 2021. This increase in turnover should be viewed in the context of the already strained health care workforce supply in many areas, such as those measured by the Health Resources and Services Administration.^26^

### Limitations

Limitations of the present study include that we were not able to identify the reason for a job flow from the data set, in particular whether a job flow occurred because of reasons relating to the firm (ie, closures and layoffs) or to the worker (ie, quitting); thus, we were unable to separately consider these 2 different types of job flows in the analysis. The J2J data also did not include finer detail on industries beyond the 2-digit NAICS codes or information on worker occupations; therefore, we were unable to examine differences in job flows across different health care settings. Given the heterogeneity of the health care workforce and findings from other studies^6,27^ reporting differing implications of the pandemic for different industries within health care, the present analysis highlights the potential benefits of publishing more granular data for industry and/or occupation categories.

## Conclusions

Using novel data on nearly all job flows in the continental US from 2018 to 2021, we quantified exit and entry rates for the health care industry before and after the COVID-19 pandemic. Our results suggest that the health care workforce experienced and is continuing to experience substantial turnover associated with the pandemic and its aftermath. Given these findings, policy efforts to address health care worker burnout and improve health care worker hiring pipelines are well warranted.

## Notes

Supplement 1. **eAppendix 1.** Details on Job Flow Definitions in Job-to-Job Flows Data and Empirical Specification **eTable 1.** Composition of NAICS Sector 62 Using Quarterly Census of Employment and Wages Data **eTable 2.** Descriptive Statistics on Health Care Workers, Exiters, and Entrants **eFigure 1.** Adjusted Average Quarterly Exit Rates from Non–Health Care Sectors **eTable 3.** Average Quarterly Health Care Exit Rates by State, 2018-2021 **eFigure 2.** Adjusted Average Quarterly Exit Rates for Health Care Workers, by Worker Education Level **eFigure 3.** Adjusted Average Quarterly Exit Rates for Health Care Workers, by Firm Size **eFigure 4.** Adjusted Average Quarterly Exit Rates for Health Care Workers, by Firm Age

Supplement 2.
Data Sharing Statement
