# Supplementary material for: Job Flows Into and Out of Health Care Before and After the COVID-19 Pandemic
Source: JAMA Health Forum. 2024 Jan 26;5(1):e234964. doi: 10.1001/jamahealthforum.2023.4964 (PMC10818214; doi:10.1001/jamahealthforum.2023.4964)
Supplement: Supplement 1. — eAppendix 1. Details on Job Flow Definitions in Job-to-Job Flows Data and Empirical Specification eTable 1. Composition of NAICS Sector 62 Using Quarterly Census of Employment and Wages Data eTable 2. Descriptive Statistics on Health Care Workers, Exiters, and Entrants eFigure 1. Adjusted Average Quarterly Exit Rates from Non–Health Care Sectors eTable 3. Average Quarterly Health Care Exit Rates by State, 2018-2021 eFigure 2. Adjusted Average Quarterly Exit Rates for Health Care Workers, by Worker Education Level eFigure 3. Adjusted Average Quarterly Exit Rates for Health Care Workers, by Firm Size eFigure 4. Adjusted Average Quarterly Exit Rates for Health Care Workers, by Firm Age [file jamahealthforum-e234964-s001.pdf]

Supplemental Online Content

Shen K, Eddelbuettel JCP, Eisenberg MD. Job flows into and out of health care before and after the COVID-19 pandemic. *JAMA Health Forum.* 2024;5(1):e234964. doi:10.1001/jamahealthforum.2023.4964

eAppendix 1. Details on Job Flow Definitions in Job-to-Job Flows Data and Empirical Specification

eTable 1. Composition of NAICS Sector 62 Using Quarterly Census of Employment and Wages Data

eTable 2. Descriptive Statistics on Health Care Workers, Exiters, and Entrants

eFigure 1. Adjusted Average Quarterly Exit Rates from Non–Health Care Sectors

eTable 3. Average Quarterly Health Care Exit Rates by State, 2018-2021

eFigure 2. Adjusted Average Quarterly Exit Rates for Health Care Workers, by Worker Education Level

eFigure 3. Adjusted Average Quarterly Exit Rates for Health Care Workers, by Firm Size

eFigure 4. Adjusted Average Quarterly Exit Rates for Health Care Workers, by Firm Age

This supplemental material has been provided by the authors to give readers additional information about their work.

eAppendix1. Details on Job Flow Definitions in Job-to-Job Flows Data and Empirical Specification

<u>Defining industry exits</u>
The J2J data create measures of exits and entrants by linking an individual with a main employer for each quarter. The main job is defined as the employer comprising the greatest combined earnings at a point-in-time. Our measure of exits uses the "Main Job Separations" measure from these data, which is a count of workers who held a beginning-of-quarter main job in the health care industry in quarter *t* and did not have any earnings from that job in time *t+1*. We separate these exits into exits into nonemployment (people who had no end-of-quarter main job with any firm in *t* or *t+1*) and exits into another sector (people who had an end-of-quarter main job in quarter *t+1*). We use the analogous measures for entries.

<u>Event-study specification</u>
Our main specification is a state-quarter level regression, where each observation represents a state s in quarter t, and n(t) is the seasonal quarter (Q1, Q2, Q3, Q4). We have 16 quarters of data (four quarters each of the four years: 2018, 2019, 2020, 2021). Our estimating equation is:

$$Y_{\{st\}} = \beta X_t + \theta_s + \lambda_{n(t)} + \epsilon_{st}$$

where X_t is a vector of indicators for each of the 12 quarters in 2019-2021 and 2018 is used as the omitted year. After obtaining the coefficients β from this regression, we calculate predicted exit or entry rates where the predictions use the average state and seasonal effects as the baseline value. Standard errors are clustered at the state level.

eTable 1. Composition of NAICS Sector 62 Using Quarterly Census of Employment and Wages Data

NAICS Code & Industry	Percent of Total 62 Industry
**621 – Ambulatory Health Services**	**44.15**
6211 – Offices of Physicians	7.86
6212 – Offices of Dentists	6.33
6213 – Offices of Other Health Practitioners	6.68
6214 – Outpatient Care Centers	7.42
6215 – Medical and diagnostic Laboratories	4.20
6216 – Home Health Care Services	5.67
6219 – Other Ambulatory Health Care Services	5.99
**622 – Hospitals**	**11.19**
6221 – General Medical and Surgical Hospitals	7.13
6222 – Psychiatric and Substance Abuse Hospitals	2.20
6223 – Other Hospitals	1.86
**623 – Nursing and Residential Care Facilities**	**16.06**
6231 – Nursing Care Facilities, Skilled Nursing	6.88
6233 – Continuing Care, Assisted Living Facilities	5.47
6239 – Other Residential Care Facilities	3.71
**624 – Social Assistance**	**28.61**
6241 – Individual and Family Services	10.72
62411 – Child & Youth Services	2.70
62412 – Services for the Elderly & Disabled	3.98
62419 – Other Individual & Family Services	4.04
6242 – Emergency and Other Relief Services	4.91
6243 – Vocational Rehabilitation Services	5.80
6244 – Child Day Care Services	7.18

eTable 2. Descriptive Statistics of Health Care Workers, Exiters, and Entrants

	All health care workers in 2020Q1, (%)	Health care exiters (2020-2021)	Health care entrants (2020-2021)
**Sex**			
Male	22.4%	23.0%	24.7%
Female	77.6%	77.0%	75.3%
**Age**			
<25	8.5%	17.8%	27.1%
25-34	23.0%	25.1%	26.0%
35-44	22.3%	18.0%	17.6%
45-54	20.6%	14.2%	13.5%
55-64	18.3%	14.2%	10.1%
65+	7.2%	10.6%	5.6%
**Race/Ethnicity**			
White, Non-Hispanic	59.0%	55.3%	53.2%
Hispanic	15.1%	13.9%	15.4%
Black, Non-Hispanic	16.7%	19.7%	18.9%
Other	9.2%	11.1%	12.5%
**Education**			
No College Degree	65.0%	61.3%	54.6%
College Degree	26.3%	20.8%	18.1%
Missing	8.6%	17.9%	27.2%

eFigure 1. Adjusted Average Quarterly Exit Rates from Non–Health Care Sectors

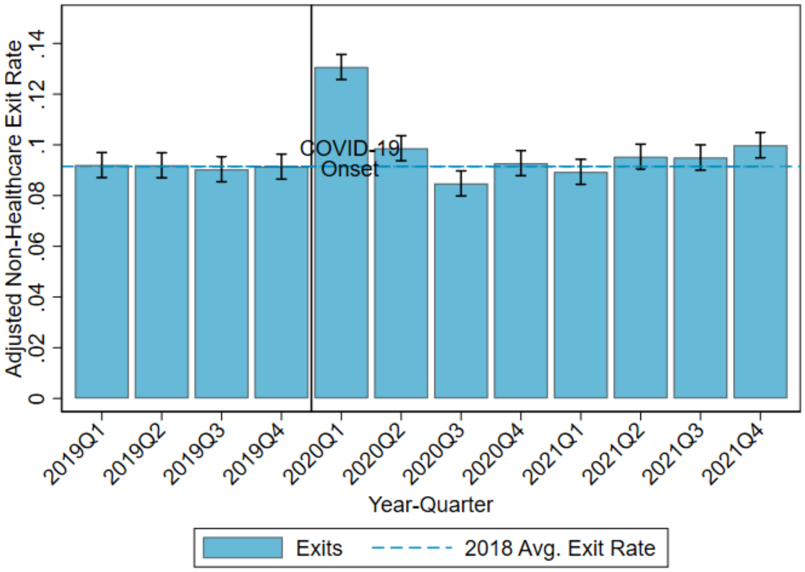

Notes: See Figure 1 in main text. We perform an analogous event study using all non–health care sectors.

eTable 3. Average Quarterly Health Care Exit Rates by State, 2018-2021

State	2018-2019	2020	2021
AL	5.87	7.09	6.85
AZ	6.35	7.32	7.27
CA	5.42	6.37	5.76
CO	7.13	8.31	9.79
CT	5.42	6.71	5.94
Washington, DC	4.96	5.61	6.22
DE	5.11	7.52	9.22
FL	6.05	6.8	7.02
GA	7.21	7.19	9.68
IA	5.38	6.18	6.4
ID	6.57	7.04	7.72
IL	5.46	6.6	6.21
IN	5.58	6.75	6.47
KS	6.06	6.54	7.45
KY	6.05	8.99	6.86
LA	6.13	6.96	7.06
MA	4.85	6.48	5.56
MD	5.35	7.61	7.38
ME	5.20	6.33	6.28
MI	5.68	7.14	5.86
MN	6.03	7.28	6.98
MO	6.41	7.09	7.54
MT	6.67	6.91	7.13
NC	5.24	5.94	6.3
ND	5.45	5.96	6.13
NE	5.63	5.99	6.53
NH	4.70	6.12	5.55
NJ	5.09	7.05	5.92
NM	7.04	7.81	7.88
NV	6.32	7.13	7.26
NY	5.38	7.07	6.29
OH	5.49	6.51	6.32
OK	6.83	7.35	7.53
OR	6.25	7.07	7.27
PA	5.20	6.26	5.93

RI	4.92	6.74	5.72
SC	5.78	6.36	6.47
SD	5.43	5.71	5.73
TX	6.72	7.28	7.33
UT	6.77	7.11	7.51
VA	5.94	7.01	6.87
VT	5.62	6.96	6.83
WA	5.79	6.29	6.64
WI	5.50	6.21	6.41
WV	5.55	6.13	6.21
WY	7.12	7.92	7.75

eFigure 2. Adjusted Average Quarterly Exit Rates for Health Care Workers, by Worker Education Level

A. No college degree B. With college degree

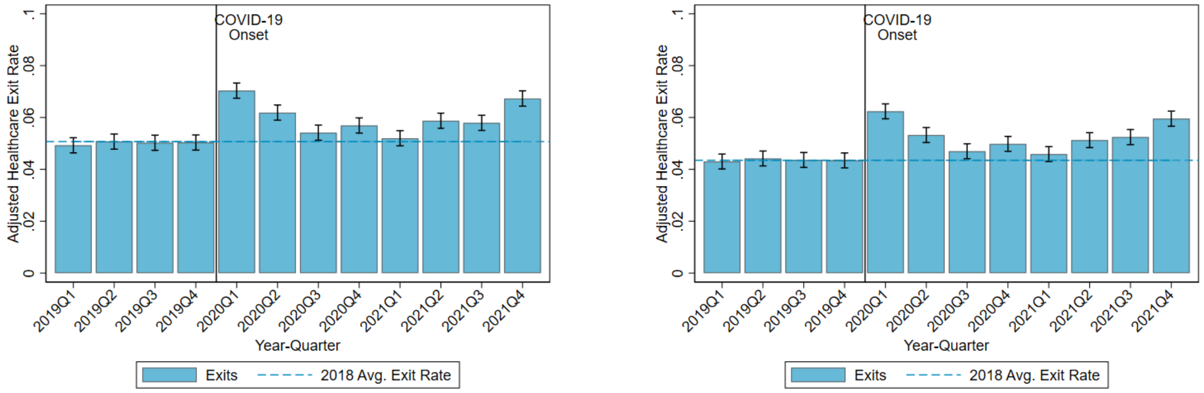

Notes: See Figure 1 in main text. We perform separate event studies for workers with and without a college degree.

eFigure 3. Adjusted Average Quarterly Exit Rates for Health Care Workers, by Firm Size

A. < 250 employees

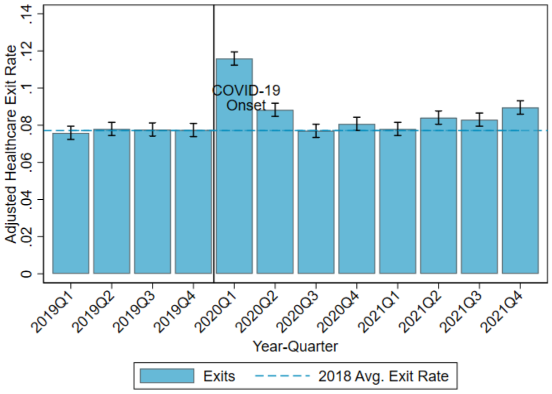

B. 250+ employees

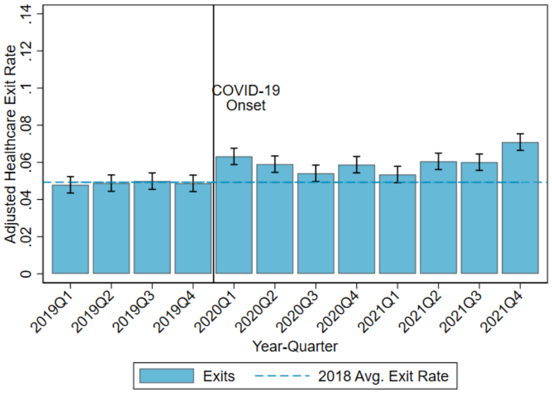

Notes: See Figure 1 in main text. We perform separate event studies for firms with fewer than 250 employees and firms with 250+ employees.

eFigure 4. Adjusted Average Quarterly Exit Rates for Health Care Workers, by Firm Age

A. 5 or less years old

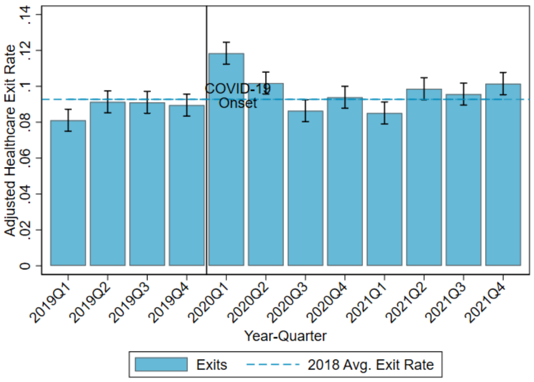

B. More than 5 years old

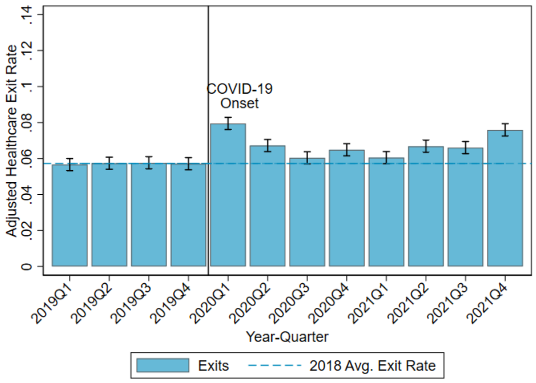

Notes: See Figure 1 in main text. We perform separate event studies for firms that are 5 or fewer years old and firms that are more than 5 years old.
