# Supplementary material for: Job Flows Into and Out of Health Care Before and After the COVID-19 Pandemic
Source: JAMA Health Forum. 2024 Jan 26;5(1):e234964. doi: 10.1001/jamahealthforum.2023.4964 (PMC10818214; doi:10.1001/jamahealthforum.2023.4964)
Supplement: Supplement 2. — Data Sharing Statement [file jamahealthforum-e234964-s002.pdf]

Data Sharing Statement

Shen. Job Flows Into and Out of Health Care Before and After the COVID-19 Pandemic. *JAMA Health Forum*. Published January 26, 2024. doi:10.1001/jamahealthforum.2023.4964

Data
Data available: Yes
Data types: Data dictionary
How to access data: The data are publicly available on the Census website. https://lehd.ces.census.gov/data/
When available: With publication

Supporting Documents
Document types: None

Additional Information
Who can access the data: Public data.
Types of analyses: Public data.
Mechanisms of data availability: Public data.
